# Patients suffering traumatic brain injury: patient characteristics, prehospital triage, primary referral and mortality - A population-based follow-up study

**DOI:** 10.1186/s13049-024-01229-7

**Published:** 2024-06-19

**Authors:** Sophie-Charlott Seidenfaden, Claus Kjaer Pedersen, Niels Juul, Hans Kirkegaard, Morten Thingemann Bøtker

**Affiliations:** 1 Research & Development, Prehospital Emergency Medical Services, Central Denmark Region, Brendstrupgårdsvej 7, Aarhus N, Denmark; 2 Department of Cardiology, Aarhus University Hospital, Palle Juul-Jensens Blvd. 99, Aarhus, Denmark; 3 Department of Anaesthesiology and Intensive Care, Section North, Neurointensive Care Unit, Aarhus University Hospital, Palle Jull-Jensens Blvd.161, Aarhus, Denmark; 4 Research Center for Emergency Medicine, Aarhus University Hospital, and Aarhus University, Palle Juul-Jensens Blvd. 99, Aarhus, Denmark; 5 Department of Clinical Medicine, Aarhus University, Incuba Skejby, bld. 2, Palle Juul-Jensens Blvd. 82, Aarhus, Denmark; 6 Department of Anaesthesiology, Randers Regional Hospital, Skovlyvej 15, Randers, Denmark

## Abstract

**Background:**

Traumatic brain injury (TBI) is a potential high-risk condition, but appropriate care pathways, including prehospital triage and primary referral to a specialised neurosurgical centre, can improve neurological outcome and survival. The care pathway starts with layman triage, wherein the patient or bystander decides whether to contact a general practitioner (GP) or emergency services (1-1-2 call) as an entryway into the health care system. The GP or 112-health care professional then decides on the level of urgency and dispatches emergency medical services (EMS) when needed. Finally, a decision is made regarding referral of the TBI patient to a specialised neurotrauma centre or a local hospital. Recent studies have shown that injuries are generally more severe in patients entering the health care system through EMS (112-calls) than through GPs; however, no information exists on whether mortality and morbidity outcomes differ depending on the referral choice. The aim of this study was to examine triage pathways, including the method of entry into the health care system, as well as patient characteristics and place of primary referral, to determine the associated 30-day and 1-year mortality rates in TBI patients with confirmed intracranial lesions.

**Methods:**

This retrospective observational population-based follow-up study was conducted in the Central Denmark Region from 1 February 2017 to 31 January 2019. We included all adult patients who contacted hospitals and were ascribed a predefined TBI ICD-10 diagnosis code in the Danish National Patient Register. The obtained TBI cohort was merged with prehospital data from the Prehospital Emergency Medical Services, Central Denmark Region, and vital status from the Danish Civil Registration System. Binary logistic regression analysis of mortality was conducted. In all patients with TBI (including concussions), the primary outcome was primary referral to a specialised centre based on mode of entry (‘GP/HCP’, ‘112-call’ or ‘Unreferred’) into the health care system. In the subgroup of patients with *confirmed* intracranial lesions, the secondary outcomes were the relative risk of death at day 30 and 1 year based on the place of primary referral.

**Results:**

Of 5,257 first TBI hospital contacts of adult patients included in the cohort, 1,430 (27.2%) entered the health care system via 1-1-2 emergency medical calls. TBI patients triaged by 112-calls were more likely to receive the highest level of emergency response (15.6% vs. 50.3%; *p* < 0.001) and second-tier resources and were more frequently referred directly to a specialised centre than were patients entering through GPs or other health care personnel. In the subgroup of 1188/5257 (22.4%) patients with *confirmed* intracranial lesions, we found no difference in the risk ratio of 30 day (RR 1.04 (95%CI 0.65–1.63)) or 1 year (RR 0.96 (95%CI 0.72–1.25)) all-cause mortality between patients primarily referred to a regional hospital or to a specialised centre when adjusting for age, sex, comorbidities, antiplatelet/anticoagulant treatment and type of intracranial lesions.

**Conclusion:**

TBI patients mainly enter the health system by contact with GPs or other health care professionals. However, patients entering through 112-calls are more frequently triaged directly to specialised centres. We were unable to demonstrate any significant difference in the adjusted 30-day and 1-year mortality based on e primary referral to a specialised centre. The inability to demonstrate an effect on mortality based on primary referral to a specialised centre may reflect a lack of clinical data in the registries used. Considerable differences may exist in nondocumented baseline characteristics (i.e., GCS, blood pressure and injury severity) between the groups and may limit conclusions about differences in mortality. Further research providing high-quality evidence on the effect of primary referral is needed to secure early neurosurgical interventions in TBI patients.

**Supplementary Information:**

The online version contains supplementary material available at 10.1186/s13049-024-01229-7.

## Background

Traumatic brain injury (TBI) is the leading cause of death and disability in young people worldwide, as well as an increasing cause of morbidity and mortality in the European elderly [1–3]. For these reasons, correct triage and referral of TBI patients is crucial to optimise conditions for correct treatment. Despite the importance of this matter, research investigating the health care pathway of the TBI patient is limited, especially regarding morbidity and mortality. In this study, we sought to highlight the differences in the health care pathways of TBI patients in the context of a Scandinavian health care system.

In Scandinavia, patients can enter the health care system by contacting a general practitioner (GP)/out-of-hours physician service or by a medical emergency call (e.g., 1-1-2 in Denmark) answered by health care professionals [4, 5]. Upon suffering a head trauma and potential TBI, patients or bystanders perform an initial self-triage when evaluating whether they need help from health care services and to which extent and urgency. The next level of triage is performed when the GPs or 112-health care professionals decide on the level of urgency and dispatch an emergency medical services (EMS) response when needed [4, 5]. While we do know that patients entering the health care system through the EMS (112-calls) are generally more severely injured than are patients entering through a GP/out-of-hours physician service, no information exists on whether a referral through an EMS results in different outcomes in mortality and morbidity than referral through a GP [6, 7]. In the case of TBI, triage is challenging due to the dynamic pathophysiology and sometimes vague initial clinical symptoms [6]. These challenges are sought to be resolved by the use of triage tools, such as the Glasgow Coma Scale (GCS) score and Injury Severity Score (ISS), to quantify symptoms [8–10]. However, these tools have their own difficulties in terms of the on-scene reliability of the TBI diagnosis and can result in over-triage in some cases [11, 12], and under-triage in others: This is especially the case in the elderly, who may initially present mild TBI symptomatology despite underlying severe TBI pathophysiology [13, 14].

Correct triage is important for several reasons: Head trauma causing intracranial damage is a high-risk condition, and primary referral to a specialised neurosurgical centre is associated with improved neurological outcome and survival [15–17]. Thus, accurate and timely identification of TBI patients expected to benefit from prehospital critical care and their direct triage to a specialised centre are considered essential elements to ensure optimal patient outcomes [4]. Furthermore, professional triage performed by GPs or 112-health care professionals should aim to limit mis-triage, including limiting over-triage of mild TBI, by evening out the differences in referral patterns induced by patient-performed self-triage when deciding on mode of entrance into the health care system.

The aim of the current study was to highlight important differences in TBI patients when comparing GP and EMS referrals. By analysing patients with either mild (ICD-10 diagnosis ‘Concussion’) or severe (all ICD-10 diagnosis indicative of intracranial pathology) TBI, we sought to1) describe patient characteristics and their mode of entrance into the health care system, 2) examine the association between mode of entrance into the health care system and primary referral to specialised centre, and 3) examine the association between primary referral to a specialised centre and the 30-day and 1-year mortality in patients with confirmed intracranial lesions.

## Methods

### Study design

This register-based retrospective, observational, population-based follow-up study was conducted in the Central Denmark Region during a 2-year period from 1 February 2017 to 31 January 2019. The results are reported according to the Strengthening the Reporting of Observational Studies in Epidemiology (STROBE) guideline [18].

### Setting

The study was conducted in the Central Denmark Region with a catchment population of 1.3 million people that accounts for 23% of the total Danish population. The Danish National Health Service is a tax-supported system and health care services are free of charge. The service provides health care facilities, such as GPs and prehospital EMS, as well as hospital services. The Central Denmark Region is one of five administrative health care regions in Denmark. The region houses five hospitals with emergency departments with trauma care functions, four regional hospitals without neurosurgical capacity (Randers, Viborg, Horsens and Herning) and one specialised centre with an emergency department with major trauma centre facilities and neurosurgical facilities (Aarhus University Hospital). In addition, daytime emergency clinics that attend to minor injuries are in place in Grenaa, Holstebro, Silkeborg, Skive and Ringkøbing [19].

### Prehospital triage, coordination and incident management

The Danish EMS is a two-tiered system of (1) ambulances staffed by emergency medical technicians and paramedics, and (2) physician-staffed critical care teams deployed in rapid-response vehicles and/or helicopters and available day and night. All units respond to both trauma and medical emergencies; the second tier is dispatched to suspected critical illness. Dispatch of all EMS responses, ground-based as well as airborne, are centrally coordinated from five regional Emergency Medical Communication Centres (EMCCs). Resources can be dispatched following either GP requests or layman 112-calls to the EMCC.

**Fig. 1:**
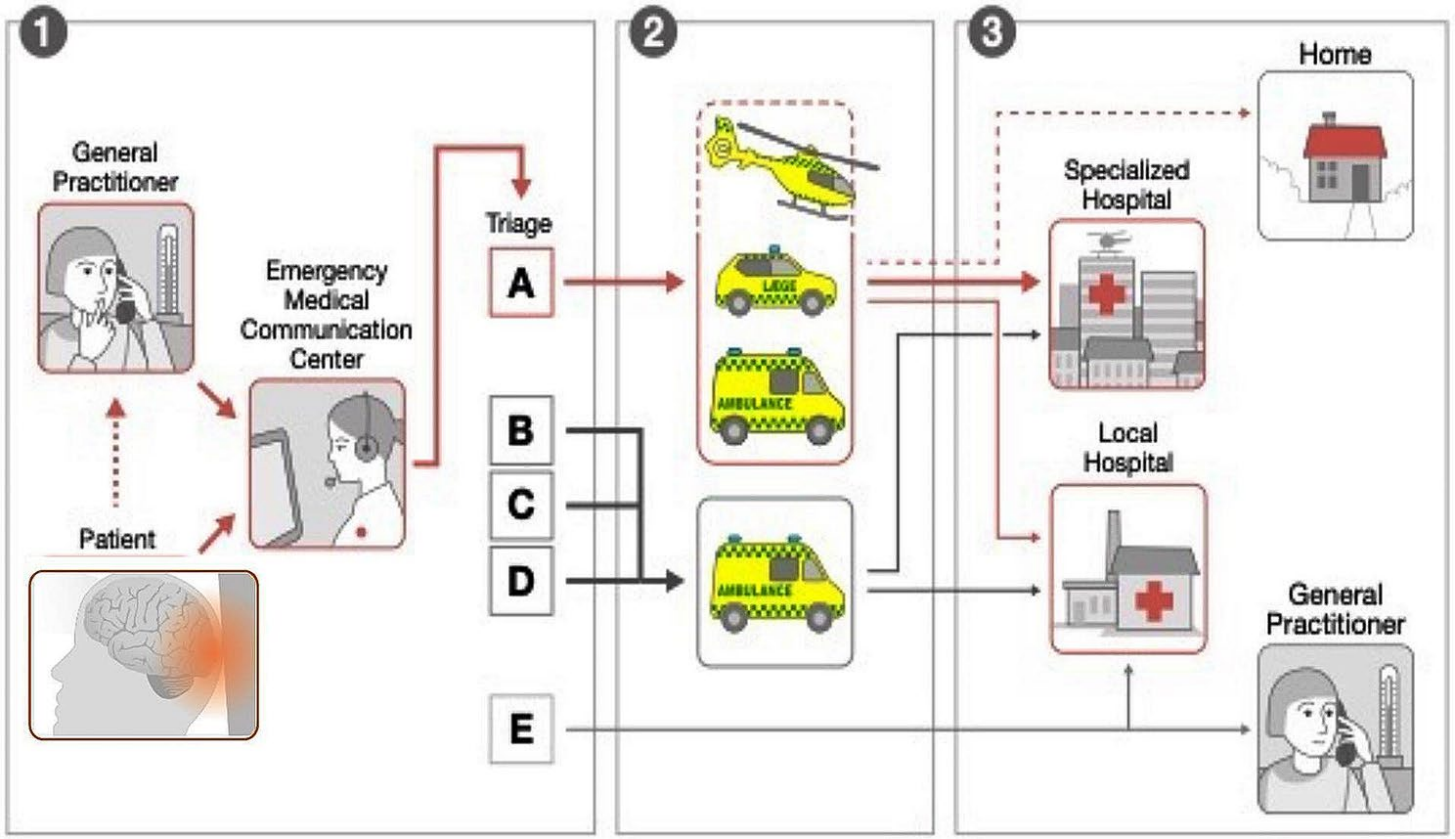
Mode of entrance. Illustration of the mode of entrance into the health care system and Emergency Medical Communication Centre triage

When GPs request prehospital dispatch, the level of emergency (see the elaboration below) and type of response is determined by the GP. The decision is based on either clinical evaluation if the patient presents to the GPs office or on telephone triage if the patient or bystander contacts the GP instead of issuing a 112 call to the EMCC in daytime hours. Triage by GPs is conducted according to guidelines related to the trauma mechanism and clinical symptoms of relevant TBI (GCS, loss of consciousness, amnesia, skull/scalp lesions, nausea/vomiting, risk factors such as age, anticoagulant treatment or alcohol/drug intoxication). In addition to the guidelines, some GPs may not abandon their own clinical discretion entirely.

**Fig. 2:**
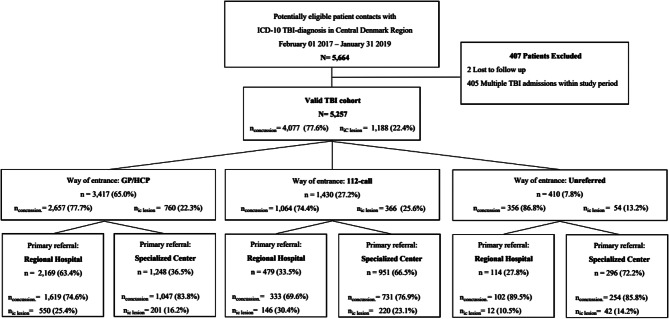
Flow diagram of TBI patients in the cohort. Inclusion and exclusion criteria and flow of emergent TBI patients through the health care system, stratified by mode of entry. Proportions are presented as total numbers in subgroups by type of TBI (concussion or intracranial lesion)

Following a layman 112-call, specially trained emergency medical technicians, paramedics, nurses and physicians in the EMCC perform a criteria-based dispatch using the criteria-based decision support tool ‘Danish Index for Emergency Care’ (Fig. 2) [5]. This triage is preformed strictly according to a list of yes/no questions based on TBI guidelines that once again contain the trauma mechanism and clinical symptoms relevant to TBI (GCS, loss of consciousness, amnesia, skull/scalp lesions, nausea/vomiting and risk factors as age, anticoagulant treatment and alcohol/drug intoxication). In cases of major injuries, the dispatch can rely solely on the type of incident rather than on a single patient level.

The level of emergency ranges from A to E (where A is lights and sirens and E is no ambulance, but oral advice or other form of service offered). Based on this classification and geography, the necessary response (ambulance and/or ground based critical care team and/or helicopter) is dispatched [5]. EMCC personnel are allowed to decline ambulance dispatch if the 112-caller is better served by another health care service. All patients must be referred by GPs or prehospital personnel in the EMCC prior to hospital visits, but patients occasionally show up unreferred. Secondary referrals from regional hospitals to Aarhus University Hospital occurs when specialised treatment is needed.

### Study population

In the present study, patients were identified through the Danish National Patient Register and comprised all adult patient (*≥* 18 years of age) contacts ascribed a predefined TBI ICD-10 diagnose code (a list of diagnoses is provided in Additional file 1, Table S4) following a hospital contact in the Central Denmark Region during the 2-year study period [20]. Contacts were then linked to prehospital data, if such contact occurred within 24 h before the TBI hospital contact. Only the first TBI hospital contact during the study period was included for each patient. Foreigners and citizens migrating within 30 days of admission were considered lost to follow-up. Follow-up was terminated on 4 April 2020.

### Data sources

All Danish citizens have a unique social security number (CPR number), which enables the linking of Danish registers on an individual level. Using CPR numbers, the TBI cohort identified through the Danish National Patient Register was merged with prehospital patient medical records and the proprietary operational dispatch database at the Prehospital EMS, Central Denmark Region. This database contains incident log numbers, dispatch criteria, response levels, timestamps, operational and patient descriptors, and general log data. Vital status at follow-up was obtained from the Danish Civil Registration System. Data on index hospital admission, and existing comorbidities were obtained from the Danish National Patient Register. Data on antiplatelet/-coagulant treatment were obtained from The Register of Pharmaceutical Sales. These Danish health registries are administered by The Danish Health Data Authority and were previously validated for research [20, 21].

### Variables

The Charlson Comorbidity Index (CCI), was calculated based on ICD-10 codes from the Danish National Patient Register from a 10-year period prior to the index contact, as originally described by Charlson et al. and validated by Thygesen et al. [22, 23]. Antiplatelet/anticoagulant treatment was reported as categorically defined by redeemed prescriptions of one of all the B01 Anatomical Therapeutic Chemical Classification system codes (ATC codes) prior to index contact: (1) acetylsalicylic acid, (2) ADP-receptor antagonist, (3) vitamin K antagonist, (4) non-vitamin K antagonist oral anticoagulant (NOAC), and (5) Other, which included unfractionated heparin and low molecular weight heparin. The mode of entry into the health care system was categorised as (1) ‘GP/Health Care Professional (HCP)’ if the hospital contact or the prehospital dispatch leading to the hospital contact was requested by a GP or another health care professional, such as nurses in nursing homes or home care nurses, (2) ‘112-call’ if the prehospital dispatch was based on an emergency medical 112-call by a layman; or (3) ‘Unreferred’ if the patient had neither prehospital nor GP contact prior to hospitalisation.

Variables describing prehospital triage and dispatch were reported as: (1) Assigned levels of initial prehospital emergency ‘A-E’, (2) Use of ambulance transport ‘yes/no’ and (3) Additional second-tier resources ‘Rapid response vehicle’ and/or ‘Helicopter’. The place of primary referral was categorised as ‘Regional hospital’ if the patient was initially admitted to one of the regional hospitals in Randers, Viborg, Horsens, or Herning and the emergency daytime clinics in Grenaa, Holstebro, Silkeborg, Skive and Ringkøbing, and as ‘Specialised Centre’ if the patient was admitted to Aarhus University Hospital. The type of TBI was dichotomised into (1) ‘Concussion’ or (2) ‘Intracranial lesion’. The specific type of intracranial lesion (epidural haemorrhage, subdural haemorrhage, subarachnoid haemorrhage, cerebral contusion and other injuries) was also reported.

### Exposures and outcomes

The primary outcome was a primary referral of the TBI patient to the specialised centre. For this primary analysis, the mode of entry (‘GP/HCP’, ‘112-call’ or ‘Unreferred’) into the health care system was regarded as exposure. In the subgroup of patients with confirmed intracranial lesions (all concussion ICD-10 codes excluded), the secondary outcomes were crude all-cause mortality and relative risks of death at days 1, 7, 30, 90 and 365. To describe the association between direct admission to a specialised centre and survival in this subgroup of patients, we performed an analysis using the place of primary referral (regional hospital or specialised centre) as exposure and mortality as outcome.

### Statistical methods

Continuous data are presented as means with 95% confidence intervals (95% CI) or medians with interquartile ranges [IQR] according to distribution. Categorical data are presented as numbers and proportions. For comparison between groups, the chi-squared test was applied for categorical data, while the two-sample t-test and Kruskal–Wallis test were used for continuous data.

Adjusted risk ratios were calculated by binary regression analysis using the specific risk ratio command with necessary transformations. The primary analysis on *all TBI cases* investigated the association between the mode of entry (GP/HCP, 112-call or unreferred) and primary referral to the specialised centre. Secondary analyses investigated the association between the place of primary referral and mortality in *TBI cases with a confirmed intracranial lesion*. In both analyses, the following covariates were adjusted: age as a continuous variable, sex as a dichotomous variable (male/female). The following categorical variables were also adjusted: CCI score, antiplatelet/anticoagulant treatment and type of intracranial lesion (epidural haemorrhage, subdural haemorrhage, subarachnoid haemorrhage, cerebral contusion and other injuries). In the sensitivity analysis, the GCS score was introduced as a categorical variable according to the stratifications of mild (GCS 14–15), moderate (GCS 9–13) and severe (GCS 3–8) TBI.

Unadjusted and adjusted mortality curves were presented using Kaplan–Meier and Cox regression curves truncated at 30 days and 1 year. All calculations were two-sided, and p-values < 0.05 were considered statistically significant. Missing data were considered missing at random and were therefore not imputed. All statistical analysis was performed using STATA© intercooled, version 17 (StataCorp LP, College Station, Texas).

## Results

### Mode of entrance

Figure 2 displays the inclusion and exclusion criteria for the cohort, as well as primary referral to either a regional hospital or the specialised centre. Of the 5,664 TBI hospital contacts made in the Central Denmark Region within the study period, 5,257 first contacts of TBI patients were included in the study cohort. Of these, 3,417/5,257 (65.0%) entered the health care system either through GP or other health care professional, whereas 1,430/5,257 (27.2%) entered by a 112-call and 410/5,257 (7.8%) showed up unreferred.

The baseline characteristics according to the mode of entry into the health care system are presented in Table 1. In general, patients entering by 112-call were older, had more comorbidities, were more often transported by ambulance, were regarded as higher levels of emergency and more often received second-tier resources when compared with patients entering by GP or other health care personnel. The proportion of patients with an intracranial lesion was higher in patients entering through 112-call (366/1,430 (25.6%)) than through GP/HCP (760/3,417 (22.3%)), *p* = 0.01 (Fig. 1). In patients with confirmed intracranial lesions, 1/3 (366/1,180 (31.0%) entered by 112-calls.

**Table 1:** Baseline characteristics, prehospital triage and direct referral of patients with TBI stratified by the mode of entrance into the health care system: General practitioner or other health care professional (GP/HCP), 112-call by layman (112-call) or unreferred, *N* = 5,257

Variable	GP/HCP *n* = 3,417 (65.0%)	112-call *n* = 1,430 (27.2%)	Unreferred *n* = 410 (7.8%)	*p*-value
**Sex**, male, n (%)	1,788 (52.3)	814 (56.9)	242 (59.0)	0.008*
**Age**, median years [IQR]	44 [18–71]	50 [24–68]	28 [20–52]	< 0.001*
**Charlson Comorbidity Index**, n (%)				
No	1,691 (49.9)	640 (44.8)	270 (65.9)	< 0.001*
Low	582 (17.0)	339 (23.7)	68 (16.7)	
Medium	580 (17.0)	272 19.0)	38 (9.3)	
High	564 (16.5)	179 (12.5)	34 (8.3)	
**Antiplatelet/-coagulant treatment**, n (%)				
Acetylsalicylic acid	327 (9.6)	152 (10.6)	26 (6.3)	
ADP-receptor antagonist	146 (4.3)	48 (3.4)	3 (0.73)	
Vitamin K antagonist	167 (4.9)	45 (3.2)	9 (2.2)	
NOAC	158 (4.6)	68 (4.8)	16 (3.9)	
Other	31 (0.9)	6 (0.4)	2 (0.49)	
**Prehospital triage and dispatch**, n (%)				
*Ambulance*, yes	1,665 (48.7)	1,299 (90.8)	0 (0.0%)	
*Level of emergency*				
A	533 (15.6)	720 (50.3)	-	< 0.001*
B	843 (24.7)	583 (40.7)	-	
C	141 (4.1)	2 (0.1)	-	
D	121 (3.5)	0 (0.0)	-	
E	5 (0.2)	3 (0.2)	-	
NA	1,774 (51.9)	122 (8.5)		
*Second-tier resources*, yes				
Rapid response vehicle (Physician)	334 (9.8)	656 (39.5)	-	< 0.001*
Rapid response vehicle (Nurse)	36 (1.1)	31 (2.2)	-	
Helicopter	26 (0.8)	48 (3.4)	-	
**Type of TBI**, n (%)				
*Concussion*	2,657 (77.7)	1,064 (74.4)	356 (86.8)	
*Intracranial lesion*	760 (22.3)	366 (25.6)	54 (13.2)	
**Direct referral to the specialised centre**, n (%)	1,248 (36.5)	951 (66.5)	296 (72.2)	< 0.001*

*Kruskal-Wallis equality-of-populations rank test

### Place of primary referral

Direct referral to the specialised centre was observed in 951/1430 (66.5%) of all patients entering by 112-calls and in 1248/3417 (36.2%) of all patients entering through GPs/HCP (risk ratio (RR) 1.65 (95%CI 1.56–1.74). In the subgroup of patients with confirmed intracranial lesions, 220/366 (60.1%) patients entering through 112-calls and 201/760 (26.4%) entering through GPs/HCP were triaged directly to the specialised centre (RR = 2.01 (95%CI 1.76–2.30)). In the subgroup of patients with concussions, 731/1064 (68.7%) of the patients entering through 112-calls and 1047/2657 (39.4%) of patients entering through GPs/HCP were triaged directly to the specialised centre (RR = 1.59 (95%CI 1.50–1.68)).

### Primary referral and mortality in patients with confirmed intracranial lesions

**Fig. 3:**
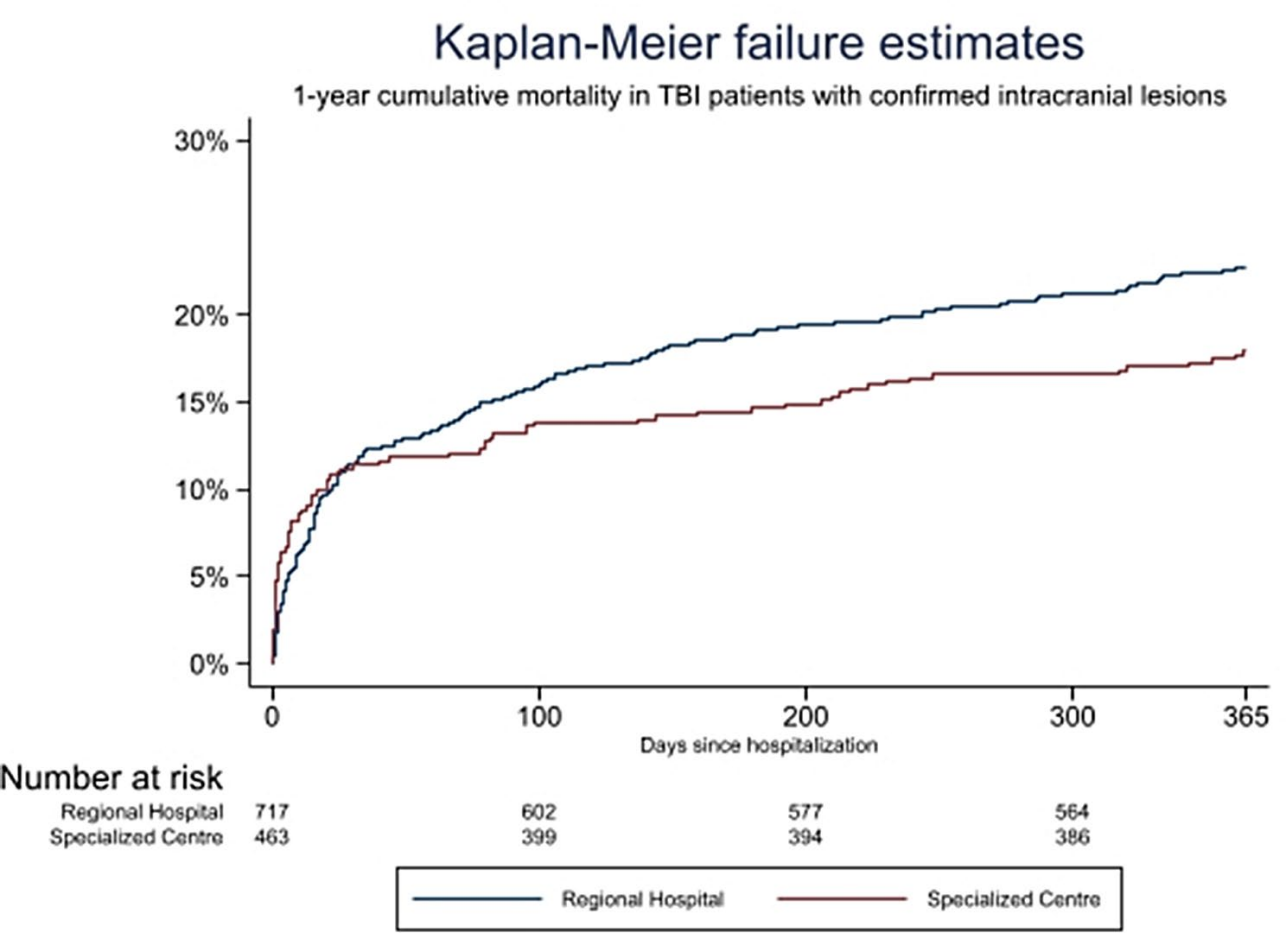
Kaplan–Meier cumulative mortality curve of long-term (1-year) all-cause mortality in TBI patients suffering from intracranial lesions, stratified by primary referral to a regional hospital or the specialised centre

**Fig. 4:**
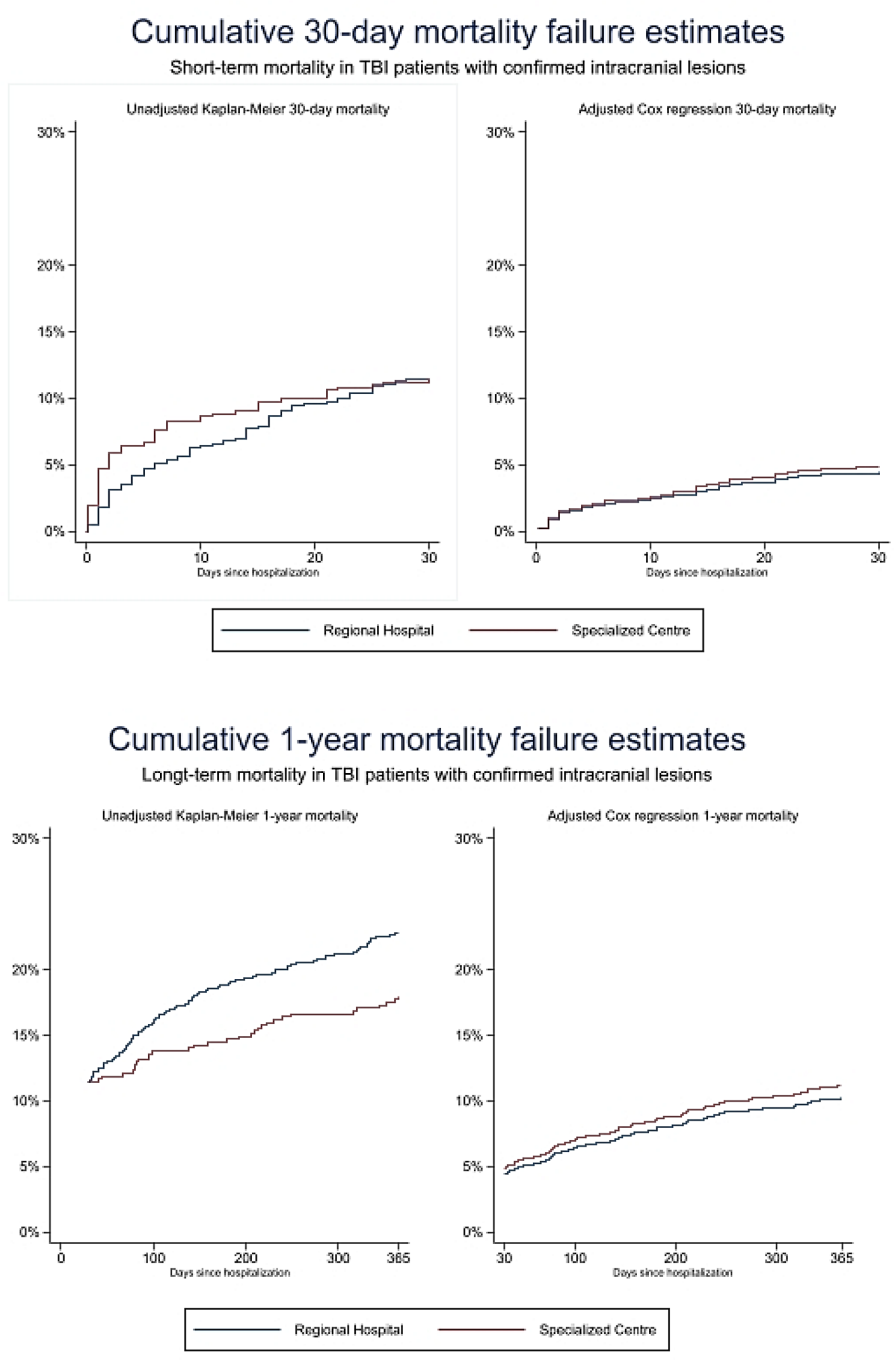
Unadjusted (Kaplan–Meier) and adjusted (Cox regression) mortality curves of short-term (30-days) and long-term (1-year) all-cause mortality in patients with TBI suffering from intracranial lesions stratified by primary referral to a regional hospital or the specialised centre

The baseline characteristics and specific types of intracranial lesions in the subgroup of patients with intracranial lesions by place of primary referral to a regional hospital and the specialised centre are given in Table 2. Crude mortality rates and unadjusted and adjusted risk ratios are presented in Table 3. The unadjusted overall 1-year mortality is presented as Kaplan–Meier cumulative mortality curves in Fig. 3. Figure 4 shows the unadjusted (Kaplan–Meier curve) and adjusted (Cox regression curve) mortality from days 0–30 and 30–365 by place of primary admission.

Among patients with intracranial lesions, those referred to the specialised centre were younger and had less comorbidity than patients referred to the regional hospitals (Table 2). We observed a higher crude early mortality in patients referred to the specialised centre than in the patients referred to the regional hospitals; day 1 RR 4.65 (95%CI 1.26–17.06) and day 7 RR 1.46 (95%CI 0.94–2.29). The number of events (death) was insufficient for valid adjusted analysis of mortality on day 1 and day 7 (Table 3).

We observed no difference in crude 30-day mortality between patients initially referred to a regional hospital at 11.4 (95%CI 9.2–13.9) and patients directly referred to the specialised centre at 11.2 (95%CI 8.5–15.5), (RR 0.98 (95%CI 0.70–1.36)). The result remained unchanged following binary regression analysis after adjusting for age, sex, comorbidity, use of antiplatelet/anticoagulant treatment and type of intracranial lesion, with RR 1.04 (95%CI 0.65–1.63) (Table 3).

In patients referred directly to the specialised centre, the crude 1-year all-cause mortality was lower, at 17.9 (95%CI 14.6–21.8) than in patients initially referred to a regional hospital, at 22.7 (95% CI 19.8–26.1) with a RR of 0.79 (0.62-1.00). When adjusting for age, sex, comorbidity, use of antiplatelet/anticoagulant treatment and type of intracranial lesion, no difference was detected, with an adjusted RR of 0.96 (95%CI 0.72–1.25) (Table 3). A sensitivity analysis conducted on the 1-year mortality but including only patients with available GCS (i.e., those transported by ambulance, *n* = 729) and adjusting for GCS instead of type of intracranial lesion (Additional file 2, Table S5), did not change the result of no difference.

**Table 2:** Baseline characteristics and type of intracranial lesions in patients with TBI with confirmed intracranial lesions stratified by primary referral to a regional or specialised centre, *N* = 1,180

Variable	Regional Hospital *n* = 717 (60.8)	Specialised Centre *n* = 463 (39.2)	*p*- value
**Sex**, male, n (%)	449 (62.6)	309 (66.7)	
**Age**, median years [IQR]	72 [56–81]	65 [45–76]	< 0.001*
**Charlson Comorbidity Index**, n (%)			
No	117 (15.9)	122 (26.4)	
Low	137 (19.1)	98 (21.2)	
Medium	228 (31.8)	143 (30.9)	
High	238 (33.2)	100 (21.6)	
**Antiplatelet/-coagulant treatment**, n (%)			
Acetylsalicylic acid	137 (19.1)	76 (16.4)	
ADP-receptor antagonist	59 (8.2)	26 (5.6)	
Vitamin K antagonist	79 (11.0)	26 (5.6)	
NOAC	50 (7.0)	33 (7.1)	
Other	15 (2.1)	7 (1.5)	
**Type of intracranial lesion**, n (%)			
Epidural haemorrhage	24 (3.4)	21 (4.5)	
Subdural haemorrhage	401 (55.9)	263 (56.8)	
Subarachnoid haemorrhage	93 (13.0)	78 (16.9)	
Cerebral Contusion	85 (11.9)	27 (5.8)	
Other	114 (15.0)	74 (16.0)	

*Two-sample T-test

**Table 3:** Crude mortality rates and risk ratios of patients with TBI with confirmed intracranial lesions by primary referral to a regional or specialised centre, *N* = 1,180

	Crude mortality rates, % (95% CI)			Risk ratios, RR (95% CI) *			
**Variable**	**Deaths**, *n*	**Regional Hospital** *n* = 717 (60.8%)	**Specialised Centre** *n* = 463 (39.2%)	**Unadjusted**	***p*** **-value**	**Adjusted**	***p*** **- value**
- 1-day	12	0.42 (0.09–1.2)	1.9 (0.9–3.7)	4.65 (1.26–17.06)	0.01	**	**
- 7-day	72	5.2 (3.7–7.0)	7.6 (5.3–10.4)	1.46 (0.94–2.29)	0.09	**	**
- 30-day	134	11.4 (9.2–13.9)	11.2 (8.5–15.5)	0.98 (0.70–1.36)	0.91	1.04 (0.65–1.63)	0.87
- 90-day	171	15.3 (12.8–18.2)	13.2 (10.2–16.6)	0.86 (0.64–1.15)	0.29	1.05 (0.73–1.5)	0.79
- 1-year	246	22.7 (19.8–26.1)	17.9 (14.6–21.8)	0.79 (0.62–1.00)	0.05	0.96 (0.72–1.25)	0.72

* Binary regression analysis, ** Insufficient number of events for adjustment

## Discussion

In this large, retrospective, population-based cohort study of patients with TBI, we found a significant difference in the level ‘A’ emergency response between the GP/HCP- and 112-call- groups (15.6% vs. 50.3%; *p* < 0.001). Direct referral to the specialised centre was observed in 951/1430 (66.5%) of all patients entering by 112-calls and in 1248/3417 (36.2%) of all patients entering through GPs/HCP (risk ratio (RR) 1.65 (95%CI 1.56–1.74). In the subgroup of patients with *confirmed* intracranial lesions, 220/366 (60.1%) patients entering through 112-call and 201/760 (26.4%) entering through GP/HCP were triaged directly to specialised centre (RR = 2.01 (95%CI 1.76–2.30)). We found no difference in either crude or adjusted 30-day all-cause mortality rates between patients primarily referred to a regional hospital compared to the specialised centre in this subgroup. At 1 year, we found a significantly lower crude mortality in patients transported to the specialised centre. However, these patients were also younger and had less comorbidity, and the adjusted analysis showed no differences in 1-year mortality.

### Comparison to other studies

The baseline patient characteristics reported in this study are in concordance with previous studies in terms of the demographics of a median age in the range 40–60 years, male predominance and subdural haemorrhage as the most frequent intracranial lesion following head trauma [2, 24, 25].

In a living systematic review of 66 studies from 23 European countries, Brazinova et al. examined the epidemiological patterns in patients with TBI from the full severity spectrum (mild, moderate and severe) based on ICD-8, 9 and 10 diagnoses as well as clinical definitions [13]. Sundstrøm et al. reported a mortality rate ranging from 3.3 to 28.4. per 100.000 inhabitants per year from 27 individual studies. In the Nordic countries, mortality in the range 10.4–21.2 per 100.000 inhabitants per year has been reported for cases of TBI identified from ICD8-10 diagnoses, including those patients who died at the scene or before arrival at the hospital. The death rate in the Danish population was 11.5 per 100.000 inhabitants per year [26]. In the current study, we found comparable mortalities, with a crude 30-day mortality of 11.2–11.4% regardless of place of primary referral and a crude 1-year mortality of 22.7% (95%CI 19.8–26.1) in regional hospitals and 17.9% (95%CI 14.6–21.8) at the specialised centre. These mortality rates are in concordance with the findings by Lecky et al., who reported no significant difference in 30-day mortality among patients transported via the pathways to the nearest hospital vs. bypass for direct transfer to specialised centre (9.1% vs. 8.8%) [11].

We found that the proportion of patients entering by 112-call versus GPs/HCP who were actually suffering from confirmed intracranial lesions was quite similar, at 25.6% versus 22.3%. To our knowledge, no previous studies have reported the mode of entry into the health care system in patients with TBI by comparing GPs, HCP and 112-calls to the prehospital EMS and unreferred patients. However, Søvsø and colleagues, as well as Huibers et al., found that patients using the EMS as an entrance to health care services were usually more severely ill, whereas patients suffering time-critical conditions still used GPs/out-of-hours physician services as an entrance [6, 7]. We were unable to demonstrate that the 112 group was more severely ill than the GP group in our TBI cohort. This may be due to a lack of clinical data on the patients, as a difference may be apparent when evaluating other variables than those included in this study. These studies by Søvsø and Huibers were also conducted on a heterogenic patient cohort and not solely on TBI patents, which could explain the different results as well.

Regarding the mortality analysis in the subgroup of patients with *confirmed* intracranial lesions, our findings of no difference in long-term mortality according to initial transfer to different types of hospitals are compliant with the recent findings on primary referral and mortality by Sewalt and the CENTER-TBI research group. Even though we reported a significantly lower crude 1-year mortality in patients directly referred to the specialised centre, the findings seemed to be caused by demographic variations (i.e. younger patients with less comorbidity) rather than by the place of primary referral. The difference in 1-year all-cause mortality was obliterated in the adjusted analysis (adjusted RR of 0.96 (95%CI 0.77–1.20). Sewalt and colleagues also found no association between primary vs. early secondary referral to a specialised neurotrauma centre and the occurrence of secondary insults (hypoxia and hypotension) or the long-term clinical outcomes [27]. Furthermore, our results are in line with the results of a meta-analysis by Pickering et al., who demonstrated no difference in mortality following direct transport to a trauma centre versus initial triage to a local hospital in their review of 11 studies on head injury patients [28].

### Interpretation

The present study and recent research contradict the otherwise established belief that direct transport of patients with TBI to hospitals with neurosurgical facilities improves patient outcome [27]. International guidelines and previous research recommend direct transport of patients with TBI to hospitals with neurosurgical facilities to reduce time delays to treatment and incidence of death and disability as an anticipated and documented effect of specialised neurotrauma care [16, 29, 30]. We therefore expected to find improved survival rates in our patients transported to the specialised centre relative to transport to a regional hospital when adjusting for relevant confounders, but we were unable to demonstrate this. We can speculate that prehospital on-scene interventions and early local in-hospital treatments to prevent secondary insults have improved to such a degree that the beneficial effects of primary referral to a specialised neurosurgical centre have diminished [31]. This may be especially the case in a region like the Central Denmark Region, where geographical distances are rather small and prehospital EMS is highly developed with a high capacity for critical care compared to other countries. In this setting, admission to a local hospital for primary workup and CT may in fact be a reasonable choice for patients when doubt exists whether a significant intracranial lesion is present.

Identifying the patients with TBI on-scene prior to making a decision on transport pathways has been queried. Recent research is now challenging the GCS-based triage, as the GCS does not reliably diagnose patients with TBI, and least of all those with severe injury needing specialised care [11, 13, 32]. In prospective studies, this incorrect triage may cause the findings of no difference as the investigated patients are simply not relevant to the study aims. This may be why the effect of early primary referral is diluted. However, our results in the current study were generated in a retrospective cohort and were based on final in-hospital ICD-10 diagnoses; therefore, on-scene triage should not have been a problem. Nevertheless, an actual beneficial effect of primary referral to specialised treatment may actually exist that we were mistakenly unable to demonstrate. If so, this could reflect our use of a cohort mainly consisting of patients with mild TBI (concussions accounted for 77.5% (4,077/5,257) of our cases) who required specialised neurosurgical treatment, thereby diluting a potential effect of primary referral to a specialised centre. A similar interpretation was presented in the work by Lecky et al., who found that only 25% of the enrolled patients suffering from TBI included only 7% needing neurosurgery [11]. However, the cohort in the current study was recruited based on a final in-hospital TBI diagnosis and not from prehospital clinical suspicions of TBI, as in the study by Lecky et al. [11]. Our design speaks to the validity of the presented results in this current study.

Conversely, we did find a significant difference in the dispatch of level of emergency ‘A’ between the GP/HCP-group and the 112-call group (15.6% vs. 50.3%; *p* < 0.001) (Table 1). The advanced and early care provided by these prehospital units, in addition to the direct transport to the specialised centre, may have compensated for an otherwise increased mortality risk in the 112-call group, thereby resulting in no increased 1-year mortality.

In conclusion, to our knowledge, high-quality evidence is still lacking regarding the effect of primary referral to secure early neurosurgical interventions in patients with TBI.

### Perspectives

The first aim of this study was to investigate patient characteristics to create an overview of the typical patients with TBI in the Central Denmark Region and to map how the patients with TBI enter the health care system. Future research should focus on initial clinical presentations and patterns in the use of health care services prior to incidents leading to TBI to understand the patterns of self-triage among the catchment population (i.e. using GPs or 112-calls to EMCC in cases of emergency). A further effort is also needed to improve the triage tools of both GPs/EMCC and on-scene prehospital personnel, as this may have a potential beneficial effect on triage and transport patterns. The current GCS-based and symptom-based triage tools present difficulties when attempting to identify high-risk patients with TBI [11, 12]. An objective measure in the form of a point-of-care diagnostic tool could optimise early triage and improve referral patterns. This is addressed further by the author’s group in a different paper [33].

### Strengths and limitations

The main strength of this study is its register-based design, which improves the precision of the findings. The unique Danish system of social security numbers provides the possibility of linking validated registers and patient records at an individual level. The risk of selection bias is also limited due to a tax-based health care system, which ensures a genuine cohort. The completeness of data for the included covariates in the adjusted analysis is also a strength. Lastly, patients with TBI are often reported as a subgroup in general trauma research, but this study presents epidemiological data on a cohort consisting solely of trauma patients with a main ICD-10 TBI diagnosis.

Nevertheless, this study has some limitations. One is that the registries used feature a general lack of clinical data, such as injury severity score, systolic blood pressure, oxygenation and long-term patient related outcomes (such as persisting headache, reduced working hours etc.). We also were not able to adjust for initial GCS for the entire cohort, as only patients transported by ambulance were GCS scored. Patients entering by GP and assigned a lower level of emergency according to The Danish Index of Emergency Care (no ambulance or civil transport) were not GCS scored. This lack of GCS adjustment could have caused residual confounding in the mortality analysis. However, our sensitivity analysis on 1-year mortality in GCS-scored patients (i.e. those transported by ambulance) and including GCS in the regression instead of the type of intracranial lesion confirmed the result of no difference. In addition, we were not able to adjust for injury severity score either. The possibility that considerable differences could exist in the nondocumented baseline characteristics (i.e. GCS, blood pressure and injury severity) between the groups highly limits the conclusions about differences in mortality. However, these important data were not available in the registries that have been used.

Another limitation is that the lack of clinical data led us to assume that confirmed intracranial lesions could act as a proxy for severe TBI and therefore enable classification of the patients at high risk of mortality. This may have caused an underestimation of the true mortality rates. Regardless, some intracranial lesions may be minor and cause morbidity rather than mortality. No data on long-term patient-related outcomes (late complications, persistent post-concussive symptoms, adhesion to the labour market) were included in the current study; therefore, the morbidity is poorly documented. Other Danish registries contain these types of data, so this can be investigated in future studies. This lack may also cause an inability to document considerable differences in baseline characteristics for the groups (i.e. initial blood pressure, GCS and injury severity score), thereby limiting the possibilities to compare the groups.

Another limitation is that TBI is a potentially fatal condition already in the prehospital setting and not all patients reach the hospital and get an ICD-10 diagnosis indicative of TBI. Numbers on high-energy traumas from the Nordic countries presented by Steinvik et al. suggest that 85.5% of deaths to occur in the prehospital setting [34]. Pfeifer and colleagues presented similar numbers from a meta-analysis in poly-trauma patients from the western world, with prehospital mortality ranging from 53.1 to 69% [35]. We found no studies on a homogenic population of patients with TBI. The prehospital mortality presented by Steinvik and Pfeifer are not directly transferrable to patients with TBI, as their patients more often suffered from low energy trauma and falls from their own height. The numbers of patients with TBI dying before hospital admission do not enter the cohort, and the resulting possible selection of patients destined to do well may underestimate mortality of the unselected TBI population.

A further limitation is that patients might have been conveyed to the geographically closest hospital instead of the most clinically relevant hospital. Due to data technicalities and legal restrictions in the variables allowed in the used registers, no data on distances from on-scene patient pickup to hospitals were available. This is likely to be a confounder when comparing triage to regional versus specialised centres and could skew the results on primary referral.

A last limitation is that the external validity of the current study is limited to prehospital EMS systems operated under similar conditions to the Danish system with the same level of prehospital critical care.

## Conclusion

More than half (65.0%) of Danish patients with TBI enter the health care system through contact with a GP/HCP. Patients with TBI entering the healthcare system through 112-calls are more likely to be triaged to the highest level of prehospital response and are more frequently primarily referred to the specialised centre than are patients triaged by GP/HCP. We found no difference in either the crude or the adjusted 30-day all-cause mortality between patients admitted to a regional hospital or specialised centre. The crude 1-year mortality was lower in patients primarily referred to the specialised centre, but the difference vanished when adjusted for age, sex, comorbidities, use of antiplatelet/ anticoagulant treatment and type of intracranial lesion. Considerable differences may exist in nondocumented baseline characteristics (i.e. GCS, blood pressure and injury severity) between the groups, resulting in highly limited conclusions about differences in mortality. Future research providing high-quality evidence with regard to the effect of primary referral is needed to ensure early neurosurgical interventions in patients with TBI.

## Electronic supplementary material

Below is the link to the electronic supplementary material.

Supplementary Material 1

Supplementary Material 2

## Data Availability

The data sets used and analysed during the current study is available from the corresponding author upon reasonable request and with relevant permissions from Danish authorities. The data is not publicly available due to protection of personal data.

## Abbreviations

ATC: Anatomical Therapeutic Chemical Classification
CCI: Charlson Comorbidity Index
CPR: Civil Personal Number
EMCC: Emergency Medical Communication Centre
EMS: Emergency Medical Services
GP: General Practitioner
HCP: Health care professional
ICD-10: International Classification of Disease 10th edition
IQR: Interquartile Ranges
N: Number
RR: Risk Ratio
TBI: Traumatic Brain Injury
112: Emergency call number
