# Supplementary material for: Patients suffering traumatic brain injury: patient characteristics, prehospital triage, primary referral and mortality - A population-based follow-up study
Source: Scand J Trauma Resusc Emerg Med. 2024 Jun 19;32:58. doi: 10.1186/s13049-024-01229-7 (PMC11186223; doi:10.1186/s13049-024-01229-7)
Supplement: Supplementary file 1 — Supplementary Material 1 [file 13049_2024_1229_MOESM1_ESM.docx]

**Additional File 1**

| **Table 4**  Frequency of index ICD-10 diagnosis included in the cohort. N = 5,257. | | |
| --- | --- | --- |
| **ICD-10 codes** | **Description** | **Frequency, n (%)** |
| DS06.0 | Concussion (commotio cerebri) | 4070 (77.4) |
| DS06.1 | Traumatic cerebral oedema | 4 (0.08) |
| DS06.2 | Diffuse brain injury | 24 (0.46) |
| DS06.2A | Traumatic compression of brain NOS | - |
| DS06.2B | Cerebral contusion/laceration NOS | 112 (2.15) |
| DS06.3 | Focal brain injury | 30 (0.57) |
| DS06.3A | Focal cerebral laceration | - |
| DS063B | Focal cerebral contusion | - |
| DS063C | Focal traumatic intracerebral contusion | - |
| DS064 | Epidural haemorrhage | 45 (0.86) |
| DS064A | Epidural haemorrhage (traumatic) | - |
| DS065 | Traumatic subdural haemorrhage | 425 (8.08) |
| DS065A | Traumatic subdural haemorrhage NOS | - |
| DS065B | Acute Traumatic subdural haemorrhage | - |
| DS065C | Chronic Traumatic subdural haemorrhage | 253 (4.81) |
| DS066 | Traumatic subarachnoid haemorrhage | 168 (3.2) |
| DS067 | Intracranial injury with prolonged coma | 5 (0.10) |
| DS068 | Other intracranial injuries | 10 (0.19) |
| DS068A | Traumatic haemorrhage, intracranial NOS | 5 (0.10) |
| DS068B | Traumatic haemorrhage, cerebellum | 1 (0.02) |
| DS068C | Traumatic haemorrhage, cerebri NOS | 27 (0.51) |
| DS068D | Traumatic intracranial haemorrhage NOS | 63 (1.20) |
| DS068E | Traumatic haemorrhage, tentorium cerebelli | - |
| DS068F | Traumatic intracranial lesion of artery/vein | - |
| DS069 | Intracranial lesion, unspecified | 13 (0.25) |
| DT060 | Lesion of brain and cranial nerves | 2 (0.04) |
