# Supplementary material for: Patients suffering traumatic brain injury: patient characteristics, prehospital triage, primary referral and mortality - A population-based follow-up study
Source: Scand J Trauma Resusc Emerg Med. 2024 Jun 19;32:58. doi: 10.1186/s13049-024-01229-7 (PMC11186223; doi:10.1186/s13049-024-01229-7)
Supplement: Supplementary file 2 — Supplementary Material 2 [file 13049_2024_1229_MOESM2_ESM.docx]

**Additional File 2**

| **Table 5**  Sensitivity analysis of crude mortality rates and risk ratios of TBI patients with confirmed intracranial lesions adjusted for GCS instead of type of intracranial lesion, N = 729 | | | | | | | |
| --- | --- | --- | --- | --- | --- | --- | --- |
|  |  | **Crude mortality rates,** % (95%CI) | | **Risk ratios,** RR (95%CI)* | | | |
| **Variable** | **Deaths,** n | **Regional Hospital**  n = 441(60.5%) | **Specialized Center**  n = 288 (39.5%) | **Unadjusted** | ***p*- value** | **Adjusted** | ***p*- value** |
| - 1-day | 8 | 0.45 (0.05-16) | 2.0 (0.7-4.4) | 4.60 (0.93-22.60) | 0.04 | ** | ** |
| - 7-day | 50 | 5.4 (3.5-7.9) | 9.0 (5.9-12.9) | 1.66 (0.97-2.83) | 0.06 | ** | ** |
| - 30-day | 93 | 12.9 (9.9-16.4) | 12.5 (8.9-16.8) | 0.97 (0.65-1.43) | 0.86 | ** | ** |
| - 90-day | 118 | 17.0 (13.6-20.8) | 14.9 (11.0-19.5) | 0.87 (0.62-1.24) | 0.45 | ** | ** |
| - 1-year | 165 | 24.3 (20.3-28.6) | 20.2 (15.7-25.3) | 0.83 (0.63-1.10) | 0.19 | 1.09 (0.74-1.60) | 0.65 |
| * Binary regression analysis, **Insufficient number of events for valid adjustment | | | | | | | |
